# Supplementary material for: Work-life balance in physicians working in two emergency departments of a university hospital: Results of a qualitative focus group study
Source: PLoS One. 2022 Nov 14;17(11):e0277523. doi: 10.1371/journal.pone.0277523 (PMC9662716; doi:10.1371/journal.pone.0277523)
Supplement: S1 Table — (DOCX) [file pone.0277523.s001.docx]

**S1 Table. Focus group guideline questions**

1. How would you describe your current work situation (as parents) in the ED?
2. Please compare the current work situation with other workplaces in general and/or other places, where you employed before in the past.
3. What could improve the job satisfaction concerning the aspect of compatibility with family/private life?
4. Does having a child influence the working relationship with other physicians?
5. Which resources could the university support and offer to improve the compatibility of work and family/private life among physicians?
6. Which work conditions in the ED would you describe as burdensome and/or family-unfriendly?
