# Supplementary material for: Work-life balance in physicians working in two emergency departments of a university hospital: Results of a qualitative focus group study
Source: PLoS One. 2022 Nov 14;17(11):e0277523. doi: 10.1371/journal.pone.0277523 (PMC9662716; doi:10.1371/journal.pone.0277523)
Supplement: S2 Table — (DOCX) [file pone.0277523.s002.docx]

**S2 Table. Synthesized results of discussions on “Past work-life balance of emergency physicians” in sub-categories and themes**

| **Main category (MC): Past work-life balance of emergency physicians** | |
| --- | --- |
| Sub-category (SC): Past work conditions and aspects of the work environment | |
| Theme (T): Lower patient volumes | *“The patient volume is just much higher [today].” (FG2)* |
| T: Interpersonal work relationships:  (+) Stronger solidarity between physicians;  (-) Stronger stigmatization of male physicians aiming at parental leave, less possibilities for female physicians to achieve work-life balance | (+) *“Especially in emergency medicine, people (…) only stay for a specific time [during their rotation] and when a social network is then built for half a year, afterwards they all drift apart again more or less. They cannot achieve to feel something special for one another (…). But the fluctuation has generally gotten higher at the university.” (FG3)*  (-) *“In the past (…) when the child was born, it was an absurdity to stay at home as a man, even for a few days (…). It was not normal. We were already somehow progressive when we brought it up to our chief at all.” (FG3)* |
| T: Work scheduling and organization: Longer shifts, less physicians scheduled per shift, frequent overtime hours | *“(...) so duties of 36 hours, if you would now say this to a junior physician in the first year of training, he would quit immediately.” (FG4)* |
| T: More pronounced hierarchies: Less possibilities for decision-making and participation of junior physicians | *“Yes, and somehow you first have to grow into it, and in the beginning you don't have much to say, I would maybe say that in a somehow transcribed way; and the young colleagues [today] don't have that anymore.” (FG4)* |
| SC: Past physician’s mentality and behavior | |
| T: Entitlement to participation of junior physicians: Less claims regarding work hours, workload, work time models and participation in duty planning | *“The generation [today] has more demands, I would say.” (FG4)* |
| T: Lower exercise of employee rights by physicians with children: Part-time work, parental leave, child sick leave | *“That you have a right to part-time work and a right to parental leave and that these rights can be fully exploited. In my opinion, these entitlements were also available in the past but we did not consider them as serious rights.” (FG3)* |
| T: Prioritization: Prioritizing of work before family or private life | *“[Now] family comes first and that has to work out, and in second place comes the clinic. Probably that’s not wrong. But that has somewhat changed.“ (FG3)* |
| SC: Past emergency medical and university medical context | |
| T: Few organizational support structures for physicians with children | *“In the past, we did not have a father’s representative (…). I think that this [role] is quite good because there are still [male and female medical] directors that when you talk to them about parental leave, you can do it on a more objective level. They [father’s representatives] sort of give emotional support.” (FG3)* |

Note: The abbreviation “FG” assigned to each quote depicts the respective number randomly allocated to each of the four focus groups.
