# Supplementary material for: Work-life balance in physicians working in two emergency departments of a university hospital: Results of a qualitative focus group study
Source: PLoS One. 2022 Nov 14;17(11):e0277523. doi: 10.1371/journal.pone.0277523 (PMC9662716; doi:10.1371/journal.pone.0277523)
Supplement: S3 Table — (DOCX) [file pone.0277523.s003.docx]

**S3 Table. Synthesized results of discussions on “Present work-life balance of emergency physicians” in sub-categories and themes**

| **Main category (MC): Present work-life balance of emergency physicians** | |
| --- | --- |
| Sub-category (SC): Present work conditions and aspects of the work environment | |
| Theme (T): Work scheduling:  (+) More opportunities for physician participation, foresighted and fair duty planning;  (-) Insufficient staffing during night shifts | (+) *“(...) so I think we do relatively individualized duty planning (...) with a relatively long-term duty planning, so as far as possible two months in advance, where it is still foreseeable.” (FG3)*  (-)*“Whereby in the late shift (…) we are four [physicians], in the night shift we are two [physicians]. (…) So we just stay longer in case there is a lot to do because we know: “Okay, two [physicians] just cannot manage this.” (FG2)* |
| T: Work organization: Many overtime hours due to unfavorable work conditions (poor patient handovers, lack of work breaks, staff shortages) and their negative impact on physical and mental health | *“(...) and I think that [the punctual end of a shift] also depends a bit on the handover culture that is cultivated.” (FG1)*  *“But if this [overtime] then occurs over a longer period of time, it is of course also a breeding ground for burnout.” (FG4)* |
|  |  |
| T: Shift work system:  (+) Possibility to hand over patients to the following shift, facilitation of the reduction of work hours or full-time work for physicians with children;  (-) Work on weekends and on public holidays, no possibility to shorten shifts, high organizational effort regarding work-life balance, high health burden, inadequate remuneration | (+) *“Well, I have to speak for shift work now. We [partner and speaker] couldn't have both worked full time during that period if I hadn't worked shifts.” (FG1)*  (-) *“(…) the problem [is] the stress factor of free time, that is somehow also part of shift work when you’re somehow on a late or night shift, you cannot make [private] appointments. You do not see anyone anymore. People with and without a family are affected.” (FG3)* |
| T: Interpersonal work relationships between physicians with and without children:  (+) Acceptance and tolerance towards physicians with children, flexible agreements with colleagues;  (-) More filling-in and overtime work by physicians without children, different prioritization regarding work-life balance | (+) *“I have to pick up my child from daycare today-, then there would be a big, collegial cooperation (…) so that it is guaranteed that you can leave on time.” (FG1)*  (-) *“Those who, let’s say, do not have family are mostly the ones who fill in.” (FG4)* |
| SC: Present aspects of residency training | |
| T: Difficulties concerning clinical rotations and career opportunities for part-time physicians with children | *“(…) one point which concerns young colleagues with children in shift work or in our department. We can reduce [work hours] easily, but we do not get rotations after having reduced [work hours]. We are being told that rotations are only possible if you work full time.” (FG1)* |
| SC: Present physician’s mentality and behavior | |
| T: (Junior) physician attitudes and values associated with overtime work: Feeling of solidarity and responsibility towards colleagues and patients, high degree of work commitment, feeling of guilt when leaving workplace on time, tendency towards seeing overtime work as a matter of fact among senior physicians | *“Maybe it is not everyone’s personality, but otherwise we wouldn't have been in the profession for such a long time, I think, because it has a certain pre-texture, right? So because of the personality structure that you just stay [for overtime].” (FG4)*  *“(...) because then with senior physicians it is also like, "It [frequent overtime] has always been like this." (FG2)* |
| T: High performance expectations regarding availability, flexibility and work effort | *“This [high work effort] is expected at 200 percent and if you can only give 150, it will be thrown at you and that is not good. So the pressure comes from the work side rather than the private side.” (FG4)* |
| T: Work experience: Lack of work experience and time management skills among junior physicians (compared to senior physicians) | *“(…) the older colleagues, they exactly know how the system works. They can, let’s say, widen the system effectively. (…) They exactly know what they can postpone.” (FG2)* |
| SC: Present emergency medical and university medical context | |
| T: High and fluctuating patient turn-over in EDs | *“I think that emergency medicine is a highly acute discipline. We never have planned patient numbers. There are days where we get 40 patients through solely in the early shift.” (FG4)* |
| T: Hospital-provided childcare:  (+) Long and flexible opening hours, extended pick-up times;  (-) Restricted availability of childcare places | *(+) “[The hospital] praises its two daycare centers with flexible and long pick-up times.” (FG1)*  *(-) “(…) and then you are graded with regard to your need and somehow [even when both parents work full-time in shifts] it was not enough to get sufficient points [to get a place in daycare].” (FG1)* |
| T: Internal clinical structures:  (+) Availability of a parent’s representative, flexible work time models;  (-) Lack of long-term contracts, poor inner-clinical patient transfer structures | (+) *“On the weekend, every child of an employee can be taken care of. Um (…) well this is a great offer.” (FG1)*  (-) *“What it can do to people when you sort of always think in periods of 12 to 24 months because then the [work] contract expires (…). Me, personally, I have felt like this for a long time and I still feel today that this is a factor of uncertainty, knowing that my contract will end someday.” (FG1)* |
| SC: ED work conditions in comparison with other (non-)clinical workplaces | |
| T: Comparison of ED sites between campuses: Less overtime work, better patient handovers, availability of a patient manager at one site | *“In [ED1] it is organized in a way where one shift lasts eight and a half hours so that it works out with three eight-hour shifts that overlap. That certainly allows for a clear scheduling while [in ED2] there is a [bigger] overlap.” (FG1)* |
| T: Other clinical workplaces compared to the ED:  (+) Clear work assignments, higher payment with less workload, strict work times and breaks, higher staffing in shifts, no reduction of physicians in the night shift, steady patient numbers, less fluctuation of staff;  (-) More chaotic scheduling, longer phases of night shifts | (+) *“For example, in disciplines where you can plan very well, like anesthesiology for example, at least also from experience, it is true that they have fixed working times, fixed breaks, where they are being replaced.” (FG4)*  (-) “*I also worked at another ward before and the duty scheduling was so bad (...) only a short time off and then early duty and then late duty and somehow everything was thrown together and also unfairly distributed.” (FG1)* |
| T: Non-clinical workplaces compared to the ED: Better break and work time regulations, less overtime work, permanent work contracts, fewer external stressors | *“There are enough workplaces where, during working hours, you can drink a coffee or just have a relaxed meal. In the hospital, however, it’s just not like that.” (FG2)* |
|  |  |
| T: ED work in Scandinavia compared to study country: Less overtime work, more work-life balance, higher prioritization of family, better payment, larger pool of substitute workers, additional vacation days for parents, better inner-clinical patient transfer structures | *“What I still can think of is the example of Denmark or Sweden (…) where the whole country is just built differently concerning the compatibility of work and family, because family is always the top priority, and it’s not like that in any field in [study country].” (FG3)* |

Note: The abbreviation “FG” assigned to each quote depicts the respective number randomly allocated to each of the four focus groups.
