# Supplementary material for: Work-life balance in physicians working in two emergency departments of a university hospital: Results of a qualitative focus group study
Source: PLoS One. 2022 Nov 14;17(11):e0277523. doi: 10.1371/journal.pone.0277523 (PMC9662716; doi:10.1371/journal.pone.0277523)
Supplement: S4 Table — (DOCX) [file pone.0277523.s004.docx]

**S4 Table. Synthesized results of discussions on “Improvement suggestions for work-life balance in emergency physicians” in sub-categories and themes**

| **Main category (MC): Improvement suggestions for work-life balance in emergency physicians** | |
| --- | --- |
| Sub-category (SC): Measures regarding work conditions and aspects of the work environment | |
| Theme (T): Work scheduling: Privileges for physicians with children (e.g., one more vacation day, one shorter shift per month), increase of the pool of part-time physicians, reduction of overlap times between shifts, increase of staffing in night shifts, adapting work schedules to current scientific standards | *“So, I would have liked it in the past if there would have been times where I could have said: “I am reducing my shift [hours] so that I can take better care of my child (…)” Like dad or mom days. That would be quite appealing.“ (FG3)* |
| T: Work organization: Reduction of overtime work (e.g., by reinforcing intermediate shifts), improving patient handovers, increasing variability in everyday work, implementation of a stricter culture of taking breaks | *“So that you don’t (…) always have 100 percent patient contact but at times you work as an emergency medical physician, at times you do the ultrasound, maybe you intubate in the OR or at times you work on the ward. This way you don’t always have the same high work demands but you get to switch [positions].” (FG2)*  *“Maybe, for example, we just have to decide as a group to take breaks.” (FG2)* |
| T: Interpersonal work relationships: Promotion of the acceptance and tolerance towards physicians with children, more frequent appraisal interviews, reinforcement of an open exchange between junior and senior physicians | *“Every year there are these [appraisal] interviews but you (…) maybe have to do them more often - in companies these interviews take place every month, with the professionals and managers. (…) every month there is an appraisal interview, especially on the management level.“ (FG4)* |
| SC: Measures regarding residency training | |
| T: Equal opportunities during training for physicians with and without children and during clinical rotations for physicians with and without work reduction | *“In my opinion, I should not have to decide between having a family or having training - or working in a hospital where I can learn a specific kind of medicine.” (FG1)* |
| SC: Measures regarding the university medical context | |
| T: Hospital-provided childcare: Increase of the number of available childcare places | *„(…)Yes, maybe more daycare places, also for doctors, (...) so those who just work.” (FG1)* |
| T: Internal clinical structures: Implementing a better inner-clinical patient transfer structure, increase of remuneration for shift workers, increase of the duration of work contracts | *“When you have the feeling that there is somehow a consistent structure that is not depending on who is on the other side [of the phone] or maybe how clearly the case is presented, then you would not tend to often call by yourself [when trying to transfer patients from the ED to wards].” (FG4)* |

Note: The abbreviation “FG” assigned to each quote depicts the respective number randomly allocated to each of the four focus groups.
