# Supplementary material for: Work-life balance in physicians working in two emergency departments of a university hospital: Results of a qualitative focus group study
Source: PLoS One. 2022 Nov 14;17(11):e0277523. doi: 10.1371/journal.pone.0277523 (PMC9662716; doi:10.1371/journal.pone.0277523)
Supplement: S1 File — (DOCX) [file pone.0277523.s005.docx]

**Consolidated criteria for reporting qualitative research (COREQ)**

Manuscript: “Work-life balance in physicians working in two emergency departments of a university hospital: Results of a qualitative focus group study”

| No, item and guide question | Page |
| --- | --- |
| Domain 1: Research team and reflexivity | |
| *Personal Characteristics* | |
| 1. Interviewer/facilitator: Which author/s conducted the interview or focus group? | 5 |
| 2. Credentials: What were the researcher’s credentials? E.g. PhD, MD | 5 |
| 3. Occupation: What was their occupation at the time of the study? | 5 |
| 4. Gender: Was the researcher male or female? | 5 |
| 5. Experience and training: What experience or training did the researcher have? | 5 |
| *Relationship with participants* | |
| 6. Relationship established: Was a relationship established prior to study commencement? | 5 |
| 7. Participant knowledge of the interviewer: What did the participants know about the researcher? e.g. personal goals, reasons for doing the research | 5 |
| 8. Interviewer characteristics: What characteristics were reported about the interviewer/facilitator? e.g. Bias, assumptions, reasons and interests in the research topic | 5 |
|  | |
| Domain 2: study design | |
| *Theoretical framework* | |
| 9. Methodological orientation and Theory: What methodological orientation was stated to underpin the study? e.g. grounded theory, discourse analysis, ethnography, phenomenology, content analysis | 8 |
| *Participant selection* | |
| 10. Sampling: How were participants selected? e.g. purposive, convenience, consecutive, snowball | 6 |
| 11. Method of approach: How were participants approached? e.g. face-to-face, telephone, mail, email | 6 |
| 12. Sample size: How many participants were in the study? | 6 |
| 13. Non-participation: How many people refused to participate or dropped out? Reasons? | 6 |
| *Setting* | |
| 14. Setting of data collection: Where was the data collected? e.g. home, clinic, workplace | 6 |
| 15. Presence of non-participants: Was anyone else present besides the participants and researchers? | 6 |
| 16. Description of sample: What are the important characteristics of the sample? e.g. demographic data, date | 7 |
| *Data collection* | |
| 17. Interview guide: Were questions, prompts, guides provided by the authors? Was it pilot tested? | 7/8 |
| 18. Repeat interviews: Were repeat interviews carried out? If yes, how many? | N/A |
| 19. Audio/visual recording: Did the research use audio or visual recording to collect the data? | 8 |
| 20. Field notes: Were field notes made during and/or after the interview or focus group? | 8 |
| 21. Duration: What was the duration of the interviews or focus group? | 7 |
| 22. Data saturation: Was data saturation discussed? | 9 |
| 23. Transcripts returned: Were transcripts returned to participants for comment and/or correction? | 8 |
|  | |
| Domain 3: analysis and findings | |
| *Data analysis* | |
| 24. Number of data coders: How many data coders coded the data? | 8 |
| 25. Description of the coding tree: Did authors provide a description of the coding tree? | 8 |
| 26. Derivation of themes: Were themes identified in advance or derived from the data? | 8/9 |
| 27. Software: What software, if applicable, was used to manage the data? | 8 |
| 28. Participant checking: Did participants provide feedback on the findings? | 8 |
| *Reporting* | |
| 29. Quotations presented: Were participant quotations presented to illustrate the themes / findings? Was each quotation identified? e.g. participant number | S2-4 Tables |
| 30. Data and findings consistent: Was there consistency between the data presented and the findings? | 9 |
| 31. Clarity of major themes: Were major themes clearly presented in the findings? | 9-18 |
| 32. Clarity of minor themes: Is there a description of diverse cases or discussion of minor themes? | 9-18 |
